# Serum 25-hydroxyvitamin D concentrations, well being, and presenteeism among hospital porters: a single-center cross-sectional study in Greece

**DOI:** 10.3389/fnut.2026.1920952

**Published:** 2026-08-28

**Authors:** Elena Vasileiou, Evangelia Nena, Paschalis Ntolios, Athanasios Zissimopoulos, Theodoros Constantinides

**Affiliations:** 1 Laboratory of Hygiene and Environmental Protection, Medical School, Democritus University of Thrace, Alexandroupolis, Greece; 2 Department of Occupational Medicine, University General Hospital of Alexandroupolis, Alexandroupolis, Greece; 3 Laboratory of Social Medicine, Medical School, Democritus University of Thrace, Alexandroupolis, Greece; 4 Department of Nursing, Faculty of Health Sciences, Democritus University of Thrace, Alexandroupolis, Greece; 5 Medical School, Democritus University of Thrace, Alexandroupolis, Greece

**Keywords:** body mass index, health personnel, hospital workers, mental health, presenteeism, psychological well being, shift work, vitamin D deficiency

## Abstract

Vitamin D deficiency is common among healthcare workers owing to indoor work, shift patterns, and limited sunlight, even in Mediterranean countries, yet evidence for hospital porters is scarce. This single-center, cross-sectional study assessed serum 25-hydroxyvitamin D [25(OH)D] concentrations and their unadjusted associations with psychological well being and presenteeism among hospital porters in a Greek tertiary university hospital in Alexandroupolis, Northern Greece, between March and June 2026. Of 46 eligible porters, 42 participated (91.3%). Serum 25(OH)D was measured by electrochemiluminescence immunoassay (ECLIA). Participants completed validated Greek versions of the WHO-5 Well Being Index and the Stanford Presenteeism Scale (SPS-6). Demographic, occupational, lifestyle, and clinical data, including BMI, were obtained from occupational-health records. The median 25(OH)D was 18.7 ng/mL (IQR 14.3–26.0), with 59.5% classified as deficient; mean BMI was 26.7 ± 3.7 kg/m^2^. In unadjusted analyses, 25(OH)D correlated positively with WHO-5 (rho = 0.371, *p* = 0.015) but not SPS-6, and inversely with BMI (rho = −0.465, *p* = 0.002). Surgical porters had lower 25(OH)D (*p* = 0.025) but higher BMI (*p* = 0.029) than general ward porters. Because no multivariable adjustment was possible and residual confounding cannot be excluded, these associations are exploratory and hypothesis-generating. These findings highlight the need for longitudinal research in this understudied workforce.

## Introduction

Vitamin D deficiency is increasingly recognized as a common public health issue worldwide (1). Vitamin D, or calciferol, is a crucial micronutrient whose effects extend beyond musculoskeletal health to include immune regulation, cardiometabolic risk, fatigue, and overall functional capacity (2). Despite abundant sunlight, Mediterranean countries consistently report unexpectedly high rates of vitamin D insufficiency, a pattern often described as the “Mediterranean paradox” (3), reflecting limited midday sun exposure, predominantly indoor activities, cultural sun-avoidance, and insufficient dietary intake (3).

Healthcare workers face demanding schedules, rotating shifts, prolonged indoor work, and physical and psychological stressors that may adversely affect health, well being, and work performance (4, 5). They experience disproportionately high rates of vitamin D deficiency, with prevalence estimates of 30–90% depending on location, environment, and season (6). Low vitamin D may intersect with musculoskeletal discomfort, fatigue, and reduced physical resilience, all of which are linked to work performance, productivity, and presenteeism -the degree to which health problems impair performance while an individual remains present at work (7, 8).

Hospital porters constitute an essential component of the healthcare workforce, as their role directly supports patient care and service delivery (9). However, research on occupational health among healthcare workers has largely focused on nurses and physicians (6), leaving support staff comparatively understudied. Porters may face similar, or potentially greater, risks related to limited sunlight exposure, physically demanding workload, and rotating shift schedules. A recent scoping review conducted by our team highlighted heterogeneity in study design and a notable lack of data on support staff, particularly porters, with evidence linking vitamin D status and mental well being remaining fragmented (6).

Given this context, this single-center, cross-sectional study aimed to generate baseline data on vitamin D status, general health, well being, and presenteeism among hospital porters at a tertiary hospital in Greece. This baseline cross-sectional analysis, distinct from the future longitudinal follow-up planned for the parent surveillance cohort, was prespecified in the study protocol approved by the Ethics Committee (ES13/07-07-2022) prior to any data collection. The prespecified objectives were to: (i) describe the prevalence of vitamin D deficiency in this group (ii) examine unadjusted associations between 25(OH)D concentrations and psychological well being (WHO-5) and presenteeism (SPS-6) and (iii) explore differences in 25(OH)D concentrations and BMI between surgical-ward and general-ward porters. Given the exploratory nature of the study, no formal directional hypotheses were tested.

## Methods

This single-center cross-sectional prevalence study was reported in accordance with the STROBE guidelines (statement provided in the Supplementary Table S1) for cross-sectional studies (10). It was conducted at the University General Hospital of Alexandroupolis, a tertiary teaching hospital in Northeastern Greece (latitude ≈40.8°N), a higher-latitude Mediterranean setting characterized by marked seasonal variation in solar irradiance. Recruitment, questionnaire administration, and blood sampling took place between 1 March and 1 June 2026 during routine occupational health assessments. The analysis used baseline data only from participants concurrently enrolled in an institutional occupational-health surveillance cohort intended for future longitudinal follow-up.

All hospital porters employed at the institution were invited to participate. Eligibility criteria included employment as a porter, age ≥18 years, employment for ≥6 months, ability to complete Greek-language questionnaires, and provision of written informed consent. Exclusion criteria, assessed through occupational health records and self-report, included any vitamin D supplementation, of any dose or formulation, within the preceding 6 months, severe chronic kidney disease (estimated glomerular filtration rate <30 mL/min/1.73 m^2^), significant hepatic disease (defined as diagnosed cirrhosis or chronic viral hepatitis), primary hyperparathyroidism, major acute illness at the time of assessment, and other conditions or medications known to substantially affect vitamin D metabolism, including malabsorption syndromes, active malignancy, anticonvulsants, glucocorticoids, and antiretroviral therapy. This exclusion criterion reflects the criterion that was actually applied during participant screening and selection; it was not modified after data collection or analysis. Kidney and hepatic function, along with all other exclusion criteria, were assessed through occupational health records for all 46 employed porters. No enrolled participant met any clinical exclusion criterion.

Of 46 employed porters, 42 met the eligibility criteria and participated (participation rate 91.3%). Of the four non-participants, one was on extended sick leave, two declined participation, and one had not met the minimum employment-duration requirement. No participant withdrew after enrolment. No formal sample-size calculation was performed because the study constituted an exploratory census of the near-complete porter workforce.

Following written informed consent, participants completed a structured survey including demographics, the Greek SPS-6 (11), and the WHO-5 Well Being Index (12). On the WHO-5, five items (0–5 each) yield a 0–100 score after multiplying by 4, with higher scores indicating better well being; on the SPS-6, six items (1–5) yield a 6–30 total, with higher scores indicating better work functioning (less presenteeism-related impairment). Additional demographic, occupational, lifestyle, and health data—age, sex, marital status, BMI, smoking, work schedule (rotating and night-shift exposure), comorbidities, medication, prior experience, and leisure- time physical activity- were obtained from occupational-health records. Comorbidities specifically reflected physician-documented diagnoses maintained by the hospital’s Occupational Health Service, rather than self-report. Dietary information was recorded when available. Educational level was also recorded from occupational-health records. All participants shared the same job title (hospital porter), the same minimum educational entry requirement (secondary-level education), and the same institutional pay scale, reflecting a homogeneous occupational and socioeconomic profile. BMI was calculated as kg/m^2^ and analyzed continuously. These variables were considered as descriptive covariates and potential confounders but, given the sample size, could not be entered simultaneously into a multivariable model. In particular, medication use was recorded as a covariate but was not analyzed as an independent determinant of serum 25(OH)D concentrations.

Serum 25-hydroxyvitamin D [25(OH)D] concentrations were measured by electrochemiluminescence immunoassay (ECLIA) using the Elecsys® Vitamin D Total III assay (Roche Diagnostics, Mannheim, Germany) on a cobas e601 analyzer in the hospital laboratory. The assay analytical measurement range was 3–100 ng/mL, with a limit of detection of approximately 3 ng/mL. Laboratory quality control was performed using the manufacturer’s PreciControl Vitamin D Total III control materials according to routine laboratory procedures.

All samples were collected in the morning (08:00–10:00), standardizing the window uniformly across both subgroups to reduce diurnal and pre-analytical variability. For descriptive purposes, 25(OH)D was categorized as deficiency (<20 ng/mL), insufficiency (20–29.9 ng/mL), and sufficiency (≥30 ng/mL), following thresholds commonly used in occupational and clinical research (13). These were used for descriptive purposes only and are not presented as definitive diagnostic criteria, given the ongoing debate over optimal cut-offs.

To characterize occupational context, a job-specific occupational characteristics profile was used to stratify participants. This tool was developed and is routinely used by the hospital’s Occupational Health Service as part of the institutional occupational risk assessment framework, to categorize employees according to job duties, work environment, and potential occupational hazards. In the present study, it was constructed from qualitative task descriptions and shift rosters rather than objective measurement of sunlight, stress, fatigue, or workload. Accordingly, the tool was used exclusively for descriptive job categorization and not as a quantitative exposure assessment instrument; it was not used to estimate exposure levels, derive exposure scores, or perform exposure–response analyses, and its formal validation as a quantitative exposure methodology was beyond the scope of the current study. Participants were stratified into (1) surgical-ward/operating-theater porters and (2) general-ward porters (Supplementary Tables S2, S3).

To limit bias, the entire workforce was invited (high participation reducing non-response bias), a single standardized ECLIA and fixed sampling window reduced measurement bias. Validated instruments were used for subjective outcomes, and self-reported data were cross-checked against occupational-health records. Since multivariable adjustment was not feasible, residual confounding could not be fully addressed.

Descriptive statistics summarized participant characteristics. Normality was assessed by Shapiro–Wilk. For all continuous variables [age, BMI, serum 25(OH)D, WHO-5, and SPS-6], normally distributed variables are presented as mean ± SD and compared using parametric tests, and non-normally distributed variables are presented as median (IQR) and compared using non-parametric tests, as detailed in Section 3.3. Group comparisons used an independent-samples *t*-test for BMI (normally distributed; *W* = 0.983, *p* = 0.783). For serum 25(OH)D, although the overall sample did not significantly deviate from normality (Shapiro–Wilk *W* = 0.956, *p* = 0.104), the Mann–Whitney *U* test was used as the primary group comparison, together with WHO-5 and SPS-6 (non-normal), given the small subgroup sample size (surgical porters, *n* = 14) and the limited power of formal normality tests at this sample size, with an independent-samples *t*-test performed as a confirmatory sensitivity analysis; associations used Spearman correlation, except where both variables were normally distributed, for which Pearson correlation was used. Significance was set at *p* < 0.05. No correction for multiple comparisons was applied given the exploratory design, so *p*-values are nominal and the risk of Type I error is acknowledged. Missing data were minimal (mostly complete records) and incomplete dietary data were excluded from analyses. Multivariable regression was not performed because the sample size was insufficient to support reliable estimation of models incorporating multiple potential confounders. Given the limited number of participants, adjustment for several covariates would likely have resulted in overfitting, imprecise estimates, and reduced model stability. Analyses were therefore restricted to unadjusted (bivariate) comparisons and correlations.

The protocol was approved by the IRB/Ethics Committee of the University Hospital of Alexandroupolis (ES13/07-07-2022), complied with the Declaration of Helsinki, and all participants provided written informed consent.

## Results

### Participant characteristics

Of 46 hospital porters assessed for eligibility, 42 were included and analyzed (participation rate 91.3%); four were not included (one extended sick leave, two declined participation, one insufficient employment duration), and none withdrew after enrollment, with complete outcome data for all 42 (participant flow diagram, Supplementary Figure S1). Of these, 27 (64.3%) were female and 15 (35.7%) were male. The median age was 50 years (IQR 43–58). Most participants reported rotating shifts (92.9%), with 90.5% reporting night-shift exposure. Comorbidities were present in 15 participants (35.7%), with arterial hypertension (*n* = 6) and thyroid disorders (*n* = 5) being the most common, followed by diabetes mellitus (*n* = 2). Only one participant (2.4%) reported any regular leisure-time physical activity, described as approximately 90 min of walking per week. Data were complete for all analytic variables except dietary information, which was recorded when available but was too incomplete for meaningful analysis and was therefore excluded from analyses (Section 2.3). Descriptive characteristics of the study population are summarized in Table 1.

**Table 1:** Descriptive characteristics of hospital porters included in the study (*N* = 42).

Variable	Value
Demographic characteristics	
Sex, *n* (%)	
Female	27 (64.3)
Male	15 (35.7)
Age (years), median (IQR)	**50 (43–58)**
Educational level, *n* (%)	
Secondary-level education	42 (100.0)
Occupational characteristics	
Job category, *n* (%)	
Surgical porters	14 (33.3)
General ward porters	28 (66.7)
Rotating shift work, *n* (%)	**39 (92.9)**
Night-shift exposure, *n* (%)	**38 (90.5)**
Health and lifestyle characteristics	
Comorbidities, *n* (%)	**15 (35.7)**
Regular physical activity, *n* (%)	**1 (2.4)**
BMI, kg/m^2^ (mean ± SD)	**26.7 ± 3.7**
Laboratory findings	
Serum 25(OH)D, ng/mL [median (IQR)]	**18.7 (14.3–26.0)**
Vitamin D status, *n* (%)	
Deficient (<20 ng/mL)	25 (59.5)
Insufficient (20–29.9 ng/mL)	11 (26.2)
Sufficient (≥30 ng/mL)	6 (14.3)
Questionnaire outcomes	
SPS-6, median (IQR)	**21 (19–23)**
WHO-5, median (IQR)	**52 (44–64)**

Data are presented as *n* (%), median (IQR), or mean ± SD, as appropriate. 25(OH)D, 25-hydroxyvitamin D; SPS-6, Stanford Presenteeism Scale; WHO-5, WHO-5 Well Being Index. BMI is reported as mean ± SD. Bold values indicate the principal descriptive results and key summary statistics reported for the study population.

### Vitamin D status

The median serum 25(OH)D concentration in the overall sample was 18.7 ng/mL (IQR 14.3–26.0). Overall, 25 participants (59.5%) were classified as deficient, 11 (26.2%) as insufficient, and 6 (14.3%) as sufficient. Serum 25(OH)D concentrations were normally distributed in the overall sample (Shapiro–Wilk test: *W* = 0.956, *p* = 0.104), although the distribution showed mild right skew, with the mean (20.2 ± 8.2 ng/mL) slightly exceeding the median (18.7 ng/mL). Given this mild skew and the modest subgroup sizes, job-category comparisons of 25(OH)D (Section 3.3) followed the prespecified primary (Mann–Whitney *U*) and sensitivity (*t*-test) approach described in Methods. The body mass index (BMI) was also normally distributed (Shapiro–Wilk test: *W* = 0.983, *p* = 0.783). The mean BMI was 26.7 ± 3.7 kg/m^2^, with approximately two-thirds of participants classified as overweight or obese. When stratified by sex, median 25(OH)D concentration was 18.7 ng/mL (IQR 15.8–26.3) in females and 16.5 ng/mL (IQR 13.9–26.3) in males. Among females, 63.0% were classified as deficient, 22.2% as insufficient, and 14.8% as sufficient, whereas among males the corresponding proportions were 53.3, 33.3, and 13.3%, respectively. No significant difference in serum 25(OH)D concentrations was observed between sexes (Mann–Whitney *U* test, *p* = 0.766), and the distribution of vitamin D categories did not differ significantly (Fisher–Freeman–Halton exact test, *p* = 0.898).

Comparisons between job categories (surgical porters: *n* = 14; general ward porters: *n* = 28) followed the prespecified approach described in Methods: the Mann–Whitney *U* test for 25(OH)D, WHO-5, and SPS-6; an independent-samples *t*-test for BMI (normally distributed in each subgroup: Shapiro–Wilk, surgical *p* = 0.967, general-ward *p* = 0.758); and a confirmatory sensitivity *t*-test for 25(OH)D (subgroup distributions also non-significant on Shapiro–Wilk: surgical *p* = 0.369, general-ward *p* = 0.161). Serum 25(OH)D concentrations were higher in general ward porters than in surgical porters (median (IQR): 20.30 (15.83–28.35) vs. 15.45 (9.83–19.75) ng/mL; Mann–Whitney *U* test, *U* = 112.0, *Z* = −2.241, *p* = 0.025), a difference that was also significant in the confirmatory sensitivity analysis using an independent-samples *t*-test (22.20 ± 7.90 vs. 16.10 ± 7.35 ng/mL; *t*(40) = 2.414, *p* = 0.020). BMI was higher in surgical porters than in general ward porters (28.47 ± 4.11 vs. 25.83 ± 3.27 kg/m^2^; independent-samples *t*-test, *t*(40) = 2.258, *p* = 0.029). Serum 25(OH)D and BMI were themselves inversely correlated (see Section 3.5); accordingly, the job-category difference in 25(OH)D and the job-category difference in BMI cannot be interpreted as independent of one another. For WHO-5, general ward porters showed higher mean ranks than surgical porters (24.04 vs. 16.43); this difference did not reach statistical significance, although a trend was observed (*U* = 125.0, *Z* = −1.914, *p* = 0.056). No statistically significant difference was observed in SPS-6 scores between job groups (mean ranks 21.86 vs. 20.79; *U* = 186.0, *Z* = −0.272, *p* = 0.786). Associations between serum 25(OH)D concentrations and demographic and clinical variables are shown in Table 2.

**Table 2:** Group comparisons by job category.

Variable	Surgical porters	General ward porters	Test statistic	df	*p*-value
Serum 25(OH)D, ng/mL [median (IQR)]	15.45 (9.83–19.75)	20.30 (15.83–28.35)	*U* = 112.0, *Z* = −2.241 (*r* = 0.43)	–	**0.025**
BMI, kg/m^2^ (mean ± SD)	28.47 ± 4.11	25.83 ± 3.27	*t* = 2.258 (*d* = 0.74)	40	**0.029**
WHO-5 score [median (IQR)]	48 (43–52)	64 (44–72)	*U* = 125.0, *Z* = −1.914 (*r* = 0.36)	–	0.056
SPS-6 score [median (IQR)]	20 (19–23)	22 (18–23)	*U* = 186.0, *Z* = −0.272 (*r* = 0.05)	–	0.786

Data are presented by job category. Descriptive values are reported as median (IQR) for non-normally distributed variables and mean ± SD for normally distributed variables. Mann–Whitney *U* tests (*U*, *Z*) were used for WHO-5 and SPS-6, which were not normally distributed (Shapiro–Wilk, *p* < 0.05). For 25(OH)D, the Mann–Whitney *U* test was used as the primary group comparison (together with WHO-5 and SPS-6) given the small subgroup sample sizes (surgical porters, *n* = 14) and the limited power of formal normality testing at this scale, notwithstanding that neither subgroup significantly deviated from normality [Shapiro–Wilk, 25(OH)D: surgical *p* = 0.369, general-ward *p* = 0.161]. An independent-samples *t*-test for 25(OH)D was performed as a confirmatory sensitivity analysis and yielded a consistent result (*t*(40) = 2.414, *p* = 0.020); the assumption of equal variances was met [Levene’s test: 25(OH)D, *p* = 0.365]. An independent-samples *t*-test was used for BMI, which was normally distributed in each subgroup (Shapiro–Wilk, BMI: surgical *p* = 0.967, general-ward *p* = 0.758) and met the assumption of equal variances (Levene’s test: BMI, *p* = 0.255). Grubbs’ test identified no statistical outliers in serum 25(OH)D in the overall sample or within either subgroup, and the job-category difference in 25(OH)D remained statistically significant, both by Mann–Whitney *U* and by *t*-test, after excluding the highest value in each subgroup (sensitivity analysis, *p* ≤ 0.029 in all scenarios), indicating the result was not driven by extreme values. Median (IQR) is retained as the descriptive summary for 25(OH)D given its mildly skewed distribution and for consistency with its reporting elsewhere in the manuscript, and is reported alongside the mean ± SD above to support both the primary (Mann–Whitney *U*) and sensitivity (*t*-test) analyses. 25(OH)D, 25-hydroxyvitamin D; BMI, body mass index; WHO-5, WHO-5 Well Being Index; SPS-6, Stanford Presenteeism Scale; SD, standard deviation; IQR, interquartile range. Bold values indicate the principal descriptive results and key summary statistics reported for the study population.

### Normality assessment

Normality of all continuous variables [age, BMI, serum 25(OH)D, WHO-5, and SPS-6] was formally assessed using the Shapiro–Wilk test. The Shapiro–Wilk test indicated that SPS-6 (*p* = 0.011) and WHO-5 (*p* < 0.001) deviated from normality; both are presented as median (IQR) and were compared using non-parametric methods. BMI, as noted above, was normally distributed (Shapiro–Wilk, *W* = 0.983, *p* = 0.783) and is presented as mean ± SD; it was analyzed using parametric methods. Serum 25(OH)D did not significantly deviate from normality in the overall sample (Shapiro–Wilk, *W* = 0.956, *p* = 0.104); nonetheless, given its mild right skew (Section 3.2), its conventional clinical reporting as median (IQR), and the limited power of Shapiro–Wilk testing at the subgroup level (surgical porters, *n* = 14), it is presented as median (IQR) throughout, with between-group comparisons following the prespecified primary (Mann–Whitney *U*) and sensitivity (*t*-test) approach described in Methods, with results shown in Table 2. Age was non-normally distributed (Shapiro–Wilk, *W* = 0.947, *p* = 0.049) and is presented as median (IQR). Q–Q plots for all continuous variables are provided in the Supplementary Figure S2).

### Outcome measures

The median SPS-6 score was 21, indicating moderate work functioning. The median WHO-5 score was 52, indicating moderate levels of psychological well being.

### Correlation analysis

Spearman correlation analysis was conducted to examine the associations among vitamin D levels, SPS-6, WHO-5, sex, job category, and BMI (Table 3).

**Table 3:** Spearman correlation coefficients among vitamin D, presenteeism, well being, sex, job category, and BMI.

Variable pair	rho	*p*-value
Vitamin D – SPS-6	0.085	0.590
Vitamin D – WHO-5	0.371^*^	0.015 (95% CI: 0.08–0.61)
Vitamin D – Sex	−0.045	0.777
Vitamin D – Job category	0.350^*^	0.023 (95% CI: 0.05–0.59)
Vitamin D – BMI	−0.465^**^	0.002 (95% CI: −0.67 to −0.19)
SPS-6 – WHO-5	0.380^*^	0.013 (95% CI: 0.09–0.61)
SPS-6 – Sex	−0.071	0.655
SPS-6 – Job category	0.042	0.789
SPS-6 – BMI	−0.063	0.690
WHO-5 – Sex	0.153	0.333
WHO-5 – Job category	0.299	0.054
WHO-5 – BMI	−0.223	0.156
Sex – Job category	−0.105	0.506
Sex – BMI	0.059	0.708
Job category – BMI	−0.327^*^	0.034 (95% CI: −0.57 to −0.03)

Values are Spearman’s rho (rho) with corresponding two-tailed *p*-values. Sex was coded as 1 = female and 2 = male, and job category was coded as 1 = surgical porters and 2 = general ward porters; therefore, positive rho values indicate higher values among males (for sex) or general ward porters (for job category). The Mann–Whitney *U* comparison should be considered the primary analysis for between-group differences by job category, and the Spearman correlation involving job category should be regarded as a secondary, coding-dependent measure of association. Rank-biserial correlations and 95% confidence intervals for Spearman correlations are reported alongside the corresponding coefficients in this table and Table 2 where applicable. *p < 0.05; **p < 0.01, two-tailed.

Vitamin D levels were positively correlated with WHO-5 scores (rho = 0.371, *p* = 0.015) and with job category (rho = 0.350, *p* = 0.023). A moderate negative correlation was observed between vitamin D and BMI (rho = −0.465, *p* = 0.002).

A positive correlation was also observed between SPS-6 and WHO-5 (rho = 0.380, *p* = 0.013).

Job category was negatively correlated with BMI (rho = −0.327, *p* = 0.034).

No statistically significant correlations were observed between sex and any of the examined variables (all *p* > 0.05). Additionally, SPS-6 was not significantly associated with vitamin D, BMI, or job category (all *p* > 0.05). WHO-5 showed a positive association with job category (rho = 0.299), although this did not reach statistical significance (*p* = 0.054).

## Discussion

This exploratory cross-sectional study provides important insights on vitamin D status, psychological well being, presenteeism, and occupational characteristics among hospital porters, an understudied population in occupational and nutritional research (6). Evidence on this population is scarce, highlighting an important gap in the literature, with only limited prior work, mainly in the context of COVID-19 and vitamin D supplementation (14). To our knowledge, this near-complete census is the first empirical study to specifically examine vitamin D status, well being, and presenteeism in hospital porters, representing a direct follow-up to the research gap identified in our own scoping review (6). All associations are unadjusted, bivariate, and uncorrected for multiple comparisons; since age, physical activity, diet, comorbidities, sun exposure, and seasonality were not controlled for, none can be interpreted as independent or causal.

The high prevalence of suboptimal vitamin D observed in this study is consistent with evidence that healthcare workers remain at risk even in sun-rich Mediterranean countries (6, 15, 16), and echoes Greek data specifically. A large laboratory-based study at a Thessaloniki tertiary hospital, at a latitude (40.7°N) comparable to Alexandroupolis (40.8°N), reported hypovitaminosis D in roughly 73% of its sample, with a mean 25(OH)D concentration of 25.08 ng/mL (17), broadly comparable to our findings, although differences in cut-off definitions limit direct comparison. A rural Northern Greece study similarly reported high rates of insufficiency and deficiency, with obesity identified as a correlate, mirroring the inverse association between 25(OH)D and BMI observed in our cohort. Nationwide Greek data indicate deficiency and insufficiency affecting approximately 40 and 33% of the general population, respectively (17), while studies from Athens and southern/island Greece, where solar irradiance is generally higher, have reported deficiency prevalences ranging from 39.5 to 92.2% depending on the thresholds applied (18). This variability fits the so-called “Mediterranean paradox” and suggests that factors beyond regional sunlight availability, including indoor working patterns, sun-avoidance behaviors, dietary intake, and skin pigmentation, may shape vitamin D status at a national level. Given the methodological heterogeneity across available Greek studies, direct numerical comparisons should be interpreted cautiously, and larger, occupation-specific, standardized studies are needed to better distinguish occupational influences from geographic and lifestyle factors.

Serum 25(OH)D concentrations were positively associated with psychological well being, as reflected by WHO-5 scores in the bivariate analysis. As with all findings in this exploratory study, this association should be interpreted cautiously as broader occupational, behavioral and lifestyle factors may underlie both variables and were not adjusted for.

In contrast, no significant association was observed between serum 25(OH)D concentrations and presenteeism, as measured by the SPS-6 scale. This null finding may reflect the multifactorial nature of work productivity, which is influenced by a complex interplay of organizational, psychosocial, and individual factors beyond nutritional status. Alternatively, the absence of an observed association may stem from limited statistical power, restricted variability in SPS-6 scores, or a genuinely weak relationship. These findings are broadly consistent with the limited literature suggesting that any relationship between vitamin D status and work productivity is likely modest and influenced by broader occupational and psychosocial determinants (19).

A key finding of this study is the particularly low vitamin D levels observed among surgical porters compared with general ward porters. This difference is compatible with, but does not establish the possibility that occupational environment is relevant to vitamin D status. One plausible, non-causal explanation, consistent with our descriptive job-specific occupational characteristics profile, is that surgical/operating-theater porters typically work in highly controlled indoor environments, under strict infection-prevention protocols and time-critical conditions, with limited opportunity for outdoor exposure, whereas general ward porters may experience greater mobility across hospital departments and potentially more incidental light exposure. However, as workload, movement patterns, and actual sunlight exposure were not objectively measured, this explanation remains untested possibility rather than confirmed mechanism.

The high prevalence of rotating shift work and night-shift exposure in the cohort may also be relevant, as night work has been linked in the broader literature with altered behavioral patterns and reduced outdoor activity (20). However, as these factors were not directly measured in this study, any link to vitamin D status here remains speculative and requires further investigation. In addition, physical activity has likewise been associated with higher serum 25(OH)D concentrations, an effect thought to be largely attributable to outdoor exposure among physically active individuals (21). In the present cohort, however, only one participant reported regular leisure-time physical activity beyond occupational duties, leaving insufficient variability to meaningfully evaluate this relationship in our data.

Furthermore, the high prevalence of overweight and obesity also merits attention in this cohort, as increased adiposity has consistently been linked with lower circulating vitamin D concentrations (22)). In the present study, BMI was inversely correlated with vitamin D levels and differed across job groups. Nevertheless, the observed relationship should be interpreted as descriptive only, as the study was not designed to probe underlying mechanisms.

From a clinical perspective, comorbidities were present in a substantial minority of participants, most commonly arterial hypertension and thyroid disorders. Although the current study was not designed to investigate causal relationships, the coexistence of low vitamin D levels and selected chronic conditions points to a worthwhile area for further research into the interplay between nutritional status and general health in this population.

## Strengths of the study

A major strength of this study is the inclusion of more than 90% (91.3%) of the hospital’s porter workforce, providing a highly representative snapshot of this occupational group in a real-world setting. This reduces the likelihood of substantial selection bias and enhances the internal validity of the findings. The study also addresses a significant evidence gap by focusing on a rather underrepresented occupational group.

Another important strength is the use of validated psychometric instruments, namely the SPS-6 and WHO-5, combined with laboratory-based measurement of vitamin D. This allowed a multidimensional assessment of nutritional status, psychological well being, and work functioning. Vitamin D concentrations were measured using electrochemiluminescence immunoassay under standardized conditions, with all samples collected within a fixed morning time window (08:00–10:00), thereby reducing biological variability and improving measurement consistency.

The development of a job-specific occupational characteristics profile represents another methodological strength, allowing structured, descriptive characterization of occupational context and supporting the interpretation of subgroup differences. As was previously stated, this tool was developed and is routinely used by the hospital’s Occupational Health Service as part of the institutional occupational risk assessment framework, and was constructed from qualitative task descriptions and shift rosters. Consistent with its descriptive purpose, it was not employed as a quantitative exposure assessment instrument.

Lastly, as a pilot study, this work provides useful preliminary data for future prospective and longitudinal research in occupational and nutritional health. The Mediterranean setting is also of particular interest in light of the so-called “Mediterranean paradox,” whereby populations living in sun-rich environments may nonetheless exhibit a high prevalence of vitamin D insufficiency or deficiency.

## Limitations of the study

This study has several limitations. The small sample size and single-center design limit statistical power and generalizability, while the cross-sectional design precludes causal inference. Accordingly, the findings should be interpreted as descriptive and hypothesis-generating. Multivariable adjustment was not feasible, leaving the observed associations susceptible to residual confounding. This limitation is particularly relevant to the job-category comparison, as BMI was higher among surgical porters and may have contributed to the observed difference in serum 25(OH)D concentrations. Medication use was similarly recorded as a covariate but was not analyzed as an independent determinant of serum 25(OH)D concentrations, and residual confounding from unmodeled medication cannot be fully excluded. Current evidence, however, does not support a clinically meaningful effect of commonly used antihypertensive agents on serum 25(OH)D; thiazide diuretics are a theoretical exception, though their documented effects relate to calcium handling and the active vitamin D metabolites rather than to serum 25(OH)D itself.

Educational level was collected and was uniform across participants (100% secondary-level education), consistent with the shared minimum entry requirement for this occupational role. Because the variable showed no variability, it could not be evaluated as a predictor or confounder. Individual-level socioeconomic status (e.g., household income) was not directly assessed. Although all participants were employed within the same institutional payroll grade, suggesting some socioeconomic homogeneity, finer-grained indicators such as tenure-related earnings differences and household characteristics were not verified.

Moreover, several established determinants of serum 25(OH)D were not comprehensively assessed, including quantified sunlight exposure, time spent outdoors, skin phototype, use of low-dose vitamin D supplements, detailed dietary intake, recent holiday or high-sunlight exposure, sleep duration and timing, and seasonal influences. Samples were collected during a period of increasing solar irradiance (March–June), and month of sampling may therefore have influenced serum 25(OH)D concentrations. However, individual sampling dates were not recorded for analysis, precluding month-stratified assessment and leaving residual seasonal confounding possible.

Dietary intake data were incomplete, and the occupational-characteristics profile was descriptive rather than instrumented. Self-reported measures may have introduced recall and social-desirability bias, likely in a non-differential manner and therefore tending to bias associations toward the null. In addition, multiple uncorrected bivariate comparisons were conducted in a small sample, increasing the risk of Type I error and potentially inflating nominal statistical significance. The study was also not powered to detect small effect sizes.

Finally, although the study achieved a near-complete census (91.3%) of one hospital’s porter workforce, enhancing internal representativeness, the findings may not generalize to other hospitals, support occupations, sunnier regions of Greece, or other healthcare systems. These issues should be addressed in larger, multicenter studies.

## Conclusion

In conclusion, low serum 25(OH)D concentrations were common in this small, single-institution sample of hospital porters. Higher serum 25(OH)D concentrations were associated with better WHO-5 well being scores, whereas no significant association was observed with SPS-6 presenteeism. Observed differences by job category require further confirmation after adjustment for BMI and other potential confounders. Due to the small sample, cross-sectional study design, and absence of multivariable adjustment, these findings should be considered preliminary and hypothesis-generating rather than causal. Accordingly, no routine screening, supplementation, nor workplace intervention is recommended on this basis. Larger, adequately powered, multicenter, and prospective studies with multivariable adjustment are needed to clarify the relationships between vitamin D status, occupational context, health, well being and productivity in this workforce, particularly in higher-latitude Mediterranean settings.

## Data Availability

The raw data supporting the conclusions of this article will be made available by the authors, without undue reservation.
