# Supplementary material for: Serum 25-hydroxyvitamin D concentrations, well being, and presenteeism among hospital porters: a single-center cross-sectional study in Greece
Source: Front Nutr. 2026 Aug 28;13:1920952. doi: 10.3389/fnut.2026.1920952 (PMC13561885; doi:10.3389/fnut.2026.1920952)
Supplement: Supplementary file 1 [file Table_1.docx]

**Supplementary**

## **Table S1.** STROBE Statement—Checklist of items that should be included in reports of cross-sectional studies

*Manuscript: Vitamin D status, well-being, and presenteeism among hospital porters at a Northern Greek tertiary hospital: a cross-sectional study*

| **Section** | **Item №** | **Recommendation** | **Reported on page/section** |
| --- | --- | --- | --- |
| Title and abstract | 1 | (a) Indicate the study's design with a commonly used term in the title or the abstract | Title/Abstract |
|  |  | (b) Provide in the abstract an informative and balanced summary of what was done and what was found | Abstract |
| **Introduction** |  |  |  |
| Background/rationale | 2 | Explain the scientific background and rationale for the investigation being reported | Introduction, para 1 |
| Objectives | 3 | State specific objectives, including any prespecified hypotheses | Introduction, para 4 |
| **Methods** |  |  |  |
| Study design | 4 | Present key elements of study design early in the paper | Methods, para 1 |
| Setting | 5 | Describe the setting, locations, and relevant dates, including periods of recruitment, exposure, follow-up, and data collection | Methods, para 1 |
| Participants | 6 | (a) Give the eligibility criteria, and the sources and methods of selection of participants | Methods, para 2 |
|  |  | (b) N/A for cross-sectional (matching criteria not applicable) |  |
| Variables | 7 | Clearly define all outcomes, exposures, predictors, confounders, and effect modifiers. Give diagnostic criteria, if applicable | Methods, paras 4-6 |
| Data sources/measurement | 8* | For each variable of interest, give sources of data and details of methods of assessment (measurement). Describe comparability of assessment methods if more than one group | Methods, paras 4-5 |
| Bias | 9 | Describe any efforts to address potential sources of bias | Methods, para 8 (bias-limitation paragraph) |
| Study size | 10 | Explain how the study size was arrived at | Methods, para 3 (census; no formal sample-size calculation) |
| Quantitative variables | 11 | Explain how quantitative variables were handled in the analyses. If applicable, describe which groupings were chosen and why | Methods, para 6 (25(OH)D categories); Methods, para 8 (stats) |
| Statistical methods | 12 | (a) Describe all statistical methods, including those used to control for confounding | Methods, para 8 |
|  |  | (b) Describe any methods used to examine subgroups and interactions | Methods, para 8 (job-category stratification) |
|  |  | (c) Explain how missing data were addressed | Methods, para 8 |
|  |  | (d) N/A (no sampling strategy beyond census) |  |
|  |  | (e) Describe any sensitivity analyses | Not applicable / none performed — confirm |
| **Results** |  |  |  |
| Participants | 13* | (a) Report numbers of individuals at each stage of study—eg numbers potentially eligible, examined for eligibility, confirmed eligible, included in the study, completing follow-up, and analysed | Results, para 1; Figure 1 (flow diagram) |
|  |  | (b) Give reasons for non-participation at each stage | Results, para 1; Figure 1 |
|  |  | (c) Consider use of a flow diagram | Figure 1 |
| Descriptive data | 14* | (a) Give characteristics of study participants (eg demographic, clinical, social) and information on exposures and potential confounders | Results, Table 1 |
| Figure s1 participant flow diagram |  | (b) Indicate number of participants with missing data for each variable of interest | Results / Table footnotes |
|  |  |  |  |

**Figure S1. Participant Flow diagram**

**Figure S2. Quantile–quantile (Q–Q) plots for continuous variables (age, body mass index, serum 25-hydroxyvitamin D, WHO-5 Well-Being Index, and Stanford Presenteeism Scale-6), used to assess normality of distribution.**

**Table S2. Job-specific occupational characteristics profile – Surgical Ward- Operating Theater Porters.**

| **Exposure Domain** | **Characteristics** |
| --- | --- |
| **Work Setting** | Operating rooms, surgical departments |
| **Work Environment** | Controlled indoor environment, artificial lighting, sterile conditions |
| **Sunlight Exposure** | Very limited (minimal outdoor exposure) |
| **Physical Workload** | High intensity; frequent patient transfers (pre/post-surgery), movement of equipment |
| **Task Type** | Time-critical patient transport, surgical logistics, equipment handling |
| **Work Rhythm** | High variability with peaks related to surgical schedules |
| **Shift Patterns** | Rotating shifts including early mornings and on-call duties |
| **Autonomy** | Low; tasks directed by surgical team priorities |
| **Psychosocial Stressors** | High time pressure, urgency, exposure to critical situations |
| **Interpersonal Interaction** | Frequent interaction with surgeons, anesthesiologists, nurses |
| **Fatigue Risk** | High due to intensity and unpredictability |
| **Infection Control Constraints** | Strict protocols (PPE, sterilization procedures) |

**Table S3. Job-specific occupational characteristics profile – General Ward Porters**

| **Exposure Domain** | **Characteristics** |
| --- | --- |
| **Work Setting** | Hospital wards, outpatient units, corridors |
| **Work Environment** | Indoor, variable conditions across departments |
| **Sunlight Exposure** | Limited but slightly higher than surgical porters (movement across hospital buildings) |
| **Physical Workload** | Moderate to high; continuous patient transport and walking |
| **Task Type** | Patient transfers between departments, logistics support |
| **Work Rhythm** | More continuous and steady workload |
| **Shift Patterns** | Rotating shifts |
| **Autonomy** | Moderate; some flexibility in task sequencing |
| **Psychosocial Stressors** | Moderate stress; workload accumulation and time demands |
| **Interpersonal Interaction** | Broad interaction with multiple departments |
| **Fatigue Risk** | Moderate (cumulative fatigue due to continuous activity) |
| **Environmental Variability** | High (different wards, noise, patient flow variability) |
